# Moving apart together: co-movement of a symbiont community and their ant host, and its importance for community assembly

**DOI:** 10.1186/s40462-021-00259-5

**Published:** 2021-05-21

**Authors:** T. Parmentier, R. Claus, F. De Laender, D. Bonte

**Affiliations:** 1 Terrestrial Ecology Unit (TEREC), Department of Biology, Ghent University, K.L. Ledeganckstraat 35, B-9000 Ghent, Belgium; 2 Research Unit of Environmental and Evolutionary Biology, Namur Institute of Complex Systems, and Institute of Life, Earth, and the Environment, University of Namur, Rue de Bruxelles 61, 5000 Namur, Belgium

**Keywords:** Ant guest, Co-dispersal, Community coexistence, Host-parasite, Inquiline, Metacommunity, Spatial structure, Succession

## Abstract

**Background:**

Species interactions may affect spatial dynamics when the movement of one species is determined by the presence of another one. The most direct species-dependence of dispersal is vectored, usually cross-kingdom, movement of immobile parasites, diseases or seeds by mobile animals. Joint movements of species should, however, not be vectored by definition, as even mobile species are predicted to move together when they are tightly connected in symbiont communities.

**Methods:**

We studied concerted movements in a diverse and heterogeneous community of arthropods (myrmecophiles) associated with red wood ants. We questioned whether joint-movement strategies eventually determine and speed-up community succession.

**Results:**

We recorded an astonishingly high number of obligate myrmecophiles outside red wood ant nests. They preferentially co-moved with the host ants as the highest densities were found in locations with the highest density of foraging red wood ants, such as along the network of ant trails. These observations suggest that myrmecophiles resort to the host to move away from the nest, and this to a much higher extent than hitherto anticipated. Interestingly, functional groups of symbionts displayed different dispersal kernels, with predatory myrmecophiles moving more frequently and further from the nest than detritivorous myrmecophiles. We discovered that myrmecophile diversity was lower in newly founded nests than in mature red wood ant nests. Most myrmecophiles, however, were able to colonize new nests fast suggesting that the heterogeneity in mobility does not affect community assembly.

**Conclusions:**

We show that co-movement is not restricted to tight parasitic, or cross-kingdom interactions. Movement in social insect symbiont communities may be heterogeneous and functional group-dependent, but clearly affected by host movement. Ultimately, this co-movement leads to directional movement and allows a fast colonisation of new patches, but not in a predictable way. This study highlights the importance of spatial dynamics of local and regional networks in symbiont metacommunities, of which those of symbionts of social insects are prime examples.

**Supplementary Information:**

The online version contains supplementary material available at 10.1186/s40462-021-00259-5.

## Background

Species interact via local and regional interactions in spatially structured networks [1, 2]. Dispersal is a central instigator of community assembly and species coexistence in these networks when it affects species interactions across space [3]. Dispersal is a three-stage process [4] comprising departure, transfer and settlement decision making. The importance of interspecific interactions has been especially documented for departure [5] and settlement [6], but it is equally important for transfer. This is particularly evident for vectored dispersal, where the transport of one species directly depends on another one, usually cross-Kingdom. Organisms or their propagules can thus be passively transported by other organisms as observed in zoochory and ectoparasitism [7, 8]. Highly advanced symbionts, for instance lichens, coral-dinoflagellate associations and some ant-symbiont associations [9–12] also passively co-disperse with their host as joint propagules.

Many organisms do not passively hitchhike, but actively follow other species guided by sensory cues provided by other species. This strategy is present in diverse groups encompassing microbes that use fungal networks as highways [13], fishes in coral reefs [14, 15] and birds that form foraging associations with other birds [16, 17] or co-forage with mammals [18]. Ultimately, these actively following organisms may co-disperse with other organisms and co-colonize new sites, and thereby have strong ecological and evolutionary implications [13, 19] for the structure and functioning of metacommunities [20]. Heterospecific attraction for instance leads to substantial deviations from predicted coexistence processes under strict competition [21].

If we aim to understand species dynamics in realistic metacommunities, we need to collect information beyond emigration probabilities and study the distance decay of movement. Such data are typically summarised in the form of the movement kernels that represent the frequency or probability distribution of movement distance in relation to the place where individuals were born and had their home range. The shape of these kernels is known to be condition dependent. That is, intraspecific interactions such as avoidance of crowding or kin competition may affect these kernels [22]. It will eventually determine the colonisation of new patches within the network, but also range expansion capacities [23]. In classic competition models, the moments of these kernels can influence the prevalence and weight of spatial coexistence mechanisms [24].

Ant nests house a diverse assemblage of arthropod species, so-called myrmecophiles [25]. These myrmecophiles span different functional groups, ranging from detritivores, scavengers, brood predators and species that prey on other myrmecophiles [26]. Ant-myrmecophile associations have been an exquisite model to study different facets of symbiosis [27, 28] and are increasingly explored in a community context [29–31]. This approach enables the comparison of disparate trait syndromes in co-habiting symbionts and are essential to understand their coexistence and the underlying community assembly rules [32]. From the perspective of the symbionts, ant nests are spatially distinct patches in a hostile environment, with age of the nest and the associated community structure determining its suitability in terms of fitness. Ant symbiont networks are thus spatially structured, and to some degree spatially heterogeneous [33, 34], opening avenues for all metacommunity dynamics to act [35]. The behaviour of myrmecophiles outside the nest and colonization events are poorly addressed. There are few anecdotal observations of myrmecophiles outside permanent ant nests [10, 36–40], but myrmecophiles are typically found in ant nests or at nest entrances. Therefore, it is generally assumed that myrmecophiles mostly reside in these nests and only leave the nest at specific events to colonize new nests [10]. Several lab studies demonstrated that myrmecophiles can follow their host by running on the chemical pheromone trails of the ant host [41–45]. Yet, it is unknown whether the trail network of the host facilitate the movement of the symbionts outside the nest and initiate co-dispersal of ants and myrmecophiles towards new nests in a natural setting. In addition to running, many myrmecophiles possess wings and may leave the nest by flying. Specific lineages of myrmecophiles such as mites may also travel outside the nest attached to the host (phoresy) and some are even carried by the host [25].

Red wood ants (*Formica rufa* group) form a group of dominant central-place foraging ants in temperate forests [46]. Their large nests contain an aboveground mound of organic thatch and a network of underground galleries [46]. Red wood ants (RWAs) move in a directed way through the landscape using trail networks. The highest densities of foraging workers outside the nest can be found on and near these trails. The trails connect the nest with trees, where they tend aphids for honeydew. Red wood ant nests may also cooperate and share resources via inter-nest trails [47]. A diverse community of arthropods lives in strict association with RWAs. These myrmecophiles are typically beetles, but other arthropod groups such as spiders and springtails are also represented [48]. Most of them live permanently in the nest, as all life stages are intranidal. We only recorded a handful of individuals outside the ant nests so far, in spite of hours of observations during the past years [49]. Other species have an alternating life cycle with one stage in the nest and the other outside the nest [49]. The main functional trophic groups that can be found in the community are predatory species that feed on other living myrmecophiles, scavengers that feed on prey and ant brood and detritivores that mainly feed on organic nest material and fungi [26].

Here, we first investigated and compared the frequency and characteristics of the mobility of macrosymbionts associated with the nests of RWAs on the forest floor. We compared the mobility of the different functional groups in the myrmecophile community. We also tested whether the symbiont community showed directed movement by co-moving with their host along the routes with the highest density of workers. Second, we studied the colonization of newly founded RWA nests by the symbionts and linked these with the observed species-specific patterns in symbiont mobility.

## Methods

### Study sites and study organisms

Our research was performed at two study sites in the North of Belgium, i.e. de Sint-Sixtusbossen, West-Vleteren (site WV, 50.885622° 2.698785°) and de Hoge Dijken, Oudenburg (site OB, 51.173453°, 3.052895°). The WV site holds a polydomous (= multiple mounds/nests) colony of *Formica rufa* Linnaeus, 1761 distributed over 48 nests (counted in 2019). Polydomous organization is widespread in red wood ants (RWAs) [46]. The polydomous colony is spread over different clusters of nests which are lined along the southern edge of deciduous forest fragments (Additional file 1: Fig. S1). Mounds in the same forest fragment cooperate, exchange food, brood and workers via trails running between the nests. Nests do not interact with nests of other forest fragments, because of physical barriers (e.g., road). Every nest mound contains multiple queens (pers. observations TP). The RWA species in OB is *Formica polyctena* Förster, 1850. *Formica rufa* and *F. polyctena* are closely related and may hybridize [50]. Their nest structure, behaviour and supported myrmecophilous fauna is similar in north west Belgium [48, 51]. The nests in OB (total of 30 nests) are more scattered as the canopy of the forest fragment is more open (Additional file 1: Fig. S1). Additionally, some nests can be found in an adjoining meadow. The social organization in the OB site is less clear than in WV. It is unknown whether the nests operate independently or exchange resources. No aggression between the mounds was recorded, but clear inter-nest trail networks are absent in this site.

### Spatial distribution of myrmecophiles outside the host nest and underlying drivers

**Fig. 1:**
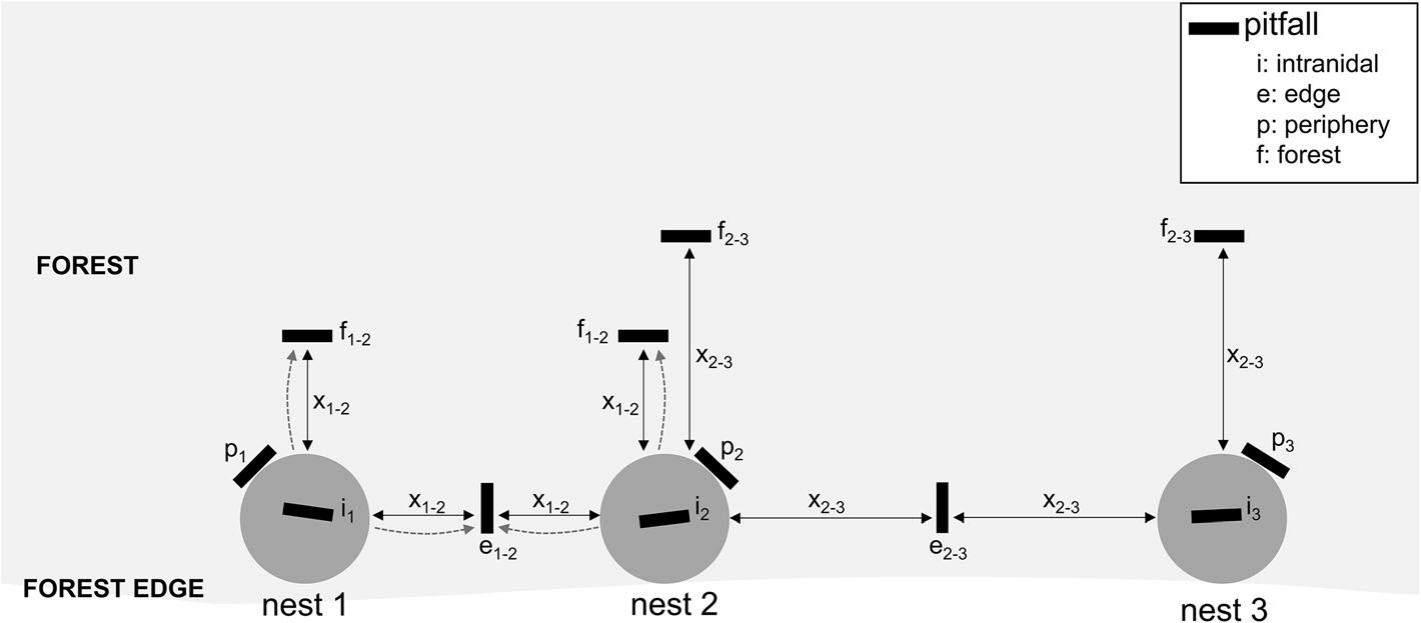
Schematic diagram of the positioning of pitfalls, here around three nests lined along a forest edge. We sampled the myrmecophiles inside a nest with an intranidal pitfall (i) and at the boundary (0 m) of a nest with a periphery pitfall (p). We placed an edge pitfall on the midpoint between two nests (along the forest edge direction). The captured myrmecophiles of this pitfall originate from either of the adjoining nests (see arrows). For both nests of this pair, a forest pitfall (f) was installed equidistant from the distance to the midpoint. Myrmecophiles found in this type of pitfall were mainly coming from the nearest nest (see single arrow). A nest which lies between two other nests in a forest fragment was part of two pairs of nests (here pair: nest1-nest2 and pair: nest2-nest3). For such a nest, two forest pitfalls were positioned at different distances: one at the half of the distance between nest 1 and 2 (midpoint distance x_1–2_), and one at the half of the distance between nest 2 and 3 (midpoint distance x_2–3_). Distance x varies from 0.6 to 25.6 m across the 20 tested nest pairs (distribution nests see Additional file 1: Fig. S1)


We assessed the spatial distribution of RWA myrmecophiles outside the nest and identified the predictors of the observed patterns. The spatial patterns were assessed using a series of pitfall traps. The densities of workers around ant nests and on the trails are extremely high, which makes classic accumulation pitfalls with a preservative not workable. Therefore, we opted for a pitfall where the ants can easily crawl out, but the myrmecophiles not. We used a plastic box (Sunware Q-Line Box: 27 × 8.4 × 9 cm, volume: 1.3 L) with a 1 cm layer of moist plaster on the bottom (Fig. S2a-c). The sides were too slippery for the myrmecophiles to escape from, but ants could easily climb out of these boxes. The rectangular pitfalls were positioned with their long side perpendicular to the direction away from the nest to maximize capture efficiency (see Fig. 1, Fig. S2b and video in Additional file 3). The pitfalls were buried so that their top rim was level with the surface of the soil. We covered pitfalls with a plastic roof to prevent rain falling in. The roof was positioned 2 cm above the opening of the pitfalls by attaching plastic caps in the corners of the roof. Soil and organic material also fell in the pitfalls (came by the wind or the ants passing by), which provided an ideal temporary habitat for the myrmecophiles (Additional file 3). This study was done entirely at the WV-site, where all nests are lined along the forest edge (Additional file 1, Fig. S1). We focused on the distribution of myrmecophiles around twenty pairs of nests formed out of 24 nests. The distance between the nests of each pair greatly varied (range: 1.2 m - 51.2 m). For each pair of nests, we installed seven pitfalls. One pitfall was placed at the midpoint between each pair of nests along the forest edge (‘edge pitfall’) (Fig. 1). These pitfalls assessed movement of myrmecophiles along the shortest path to the other nest of the pair and were often positioned on an inter-nest trail. Movement along this trajectory was expected to be the preferred direction. We compared this movement direction with the perpendicularly orientated movement away from the forest edge towards the inner forest. Therefore, we placed for each nest of the nest pair a pitfall (‘forest pitfall’) on a line segment originating from the nest and perpendicular to the shortest inter-nest path. We positioned these pitfalls in such a way that a nest was equidistant from the edge and forest pitfall (Fig. 1). Next, we positioned a pitfall just outside each nest of the nest pair (‘periphery pitfall’, periphery = 0 m). The peripheral zone was discernible from the actual nest by the lack of nest openings and organic material. These pitfalls were not aligned with the other extranidal pitfalls to avoid trapping myrmecophiles before they could reach other extranidal pitfalls. We also burrowed a pitfall inside every nest (‘intranidal pitfall’) of a focal pair of nests (Fig. 1).

**Additional file 3:** Video featuring a pitfall near a wood ant nest.

The pitfalls were left for 1 week and then emptied in a large tray in the field. Myrmecophiles were counted and identified to species (beetles following [52, 53] spiders following [54]) and also the number of *F. rufa* workers in the pitfall (including the individuals on the inner walls) was counted. RWA networks are relatively stable over the season, and therefore the number of ants in the pitfall at the time of sampling is a good proxy for the general ant activity at that location. Pitfalls were emptied and ants were counted between 11 h and 15 h to minimize effects of temperature on the activity of the ants. Pitfalls which were positioned on or near trails were visited by much more workers than pitfalls away from them. We grouped the myrmecophilous species *Monotoma angusticollis* and *Monotoma conicicollis* as *Monotoma*, because they can only be distinguished using a stereomicroscope*.* We used the same type of pitfalls with roofs to assess the diversity in the nests. As ants gradually fill the pitfall with nest material, these boxes had to be emptied sooner to avoid that the myrmecophiles could escape. We emptied these boxes every 1–2 days and kept the myrmecophiles apart to avoid double counting. After a week, we sampled these boxes a last time and the myrmecophiles that were caught during the week were put back in their nest of origin. In this way, the intranidal sampling effort was similar to the extranidal sampling effort.

The two forest and common edge pitfall were sampled three times (7-day interval between resampling), resulting in nine pitfalls per distance level. The peripheral pitfalls were also sampled three times. Sampling of peripheral, forest and edge pitfalls was organized in 9 time periods: first 5 pairs of nests were checked at 01/07, 08/07 and 15/07/2019, the following 7 pairs of nests at 22/07, 29/07 and 05/08/2019 and the last 8 pairs of nests at 12/08, 19/08 and 26/08/2019. Intranidal pitfalls were only tested once, after the third replicate of each set of nests. A total of 279 pitfalls were emptied (24 intranidal, 75 peripheral, 60 edge and 120 forest pitfalls). Ants and myrmecophiles were put back after each sampling approximately two meters from the pitfall to avoid that they would directly fall back in the pitfall. We moistened the plaster if needed and put the empty pitfall back in place and did the same sampling protocol for the next replicate.

#### Spatial distribution of myrmecophiles outside the nest

In a first analysis, we plotted the distribution of myrmecophiles (abundances and proportion of pitfalls with individuals) along the spatial gradient outside the nest. Next, we compared myrmecophiles and the different functional groups in their tendency to leave the nest, by dividing per species the average number of individuals in an extranidal pitfall (> 0 m) by the average number of individuals in a nest pitfall. The higher the ratio, the higher the tendency to leave the nest. Some myrmecophiles may often leave the nest, but stay very close to the nest entrances. To differentiate this with the tendency to leave the nest, we divided per species the average number of individuals in a peripheral pitfall (0 m) by the average number of individuals in a nest pitfall. We calculated these ratios for each time period, resulting in nine extranidal and nine peripheral estimates per species. Overall differences among the myrmecophiles in the tendency to leave the nest or to occur at the periphery were assessed using a non-parametric Kruskal-Wallis test, with myrmecophile species as independent predictor. Pairwise comparisons in the tendency to leave the nest or to occur at the periphery between the myrmecophile species were tested using Pairwise Wilcoxon Rank Sum Tests with the Benjamini-Hochberg correction for multiple testing [55].

In addition, we wanted to test whether myrmecophile species differ in their long-distance movement. For each myrmecophile species, we selected the individuals in the upper decile of the distance distribution outside the nest (periphery not included). Overall differences in long-distance movement among the top movers of the myrmecophiles were tested using a non-parametric Kruskal-Wallis test, with myrmecophile species as independent predictor. Post hoc pairwise comparisons were performed by Pairwise Wilcoxon Rank Sum Tests with the Benjamini-Hochberg correction [55].

#### Factors affecting the spatial distribution of myrmecophiles outside host nests

First, we assessed whether the distribution of individual myrmecophile species (i) is inversely related to the distance away from the nearest nest (ii) and positively affected by higher numbers of foraging RWA workers at a given distance outside the nest. The highest number of foraging workers outside the nest is found on and near trails. A positive correlation between ant activity/density and myrmecophile density outside the nest does not automatically imply that the ants affect the movement directions of the myrmecophiles. This association can be the consequence of movement in similar directions away from the nest (for example to shared food patches). However, the distribution of resources outside the nest is very homogeneous for myrmecophiles and hence no directed movement is expected. By contrast, RWAs do show very directed movement outside the nest and preferentially move towards food patches and other nests using trails [46]. In addition, many lab experiments clearly showed that myrmecophiles follow the pheromone trails of their host [41–45]. As such, we expect that the directed movement of myrmecophiles overlapping with the preferred RWA routes, can be explained by the myrmecophiles making use of the host ants and its pheromone network to move outside the nest. Note that myrmecophiles caught outside the nest are not necessarily dispersing to another nest, but may forage as well. For this first set of analyses, we only focused on the peripheral pitfalls (0 m, *N =* 75) and the forest pitfalls (*N =* 120) and did not test the directionality of movement (forest vs edge). Per myrmecophile species, we modelled number of individuals found in the pitfalls against the predictors distance from the nearest nest, density of RWA workers in the pitfall and intranidal density in the nearest nest. The latter covariate was included as the number of emigrants was expected to be positively correlated with the intranidal densities. We also included the interaction between distance and density of RWA workers as a predictor. As the models showed overdispersion, we used a negative binomial generalized linear mixed-effect model with poisson error distribution and log link function (glmer.nb function, R package lme4). The nearest nest of a pitfall and the sampling period were modelled as random factors. We ran these models for the following species: *Thyreosthenius biovatus*, *Stenus aterrimus*, *Thiasophila angulata*, *Lyprocorrhe anceps*, *Notothecta flavipes*, *Pella humeralis* and *Cyphoderus albinus*. The other species were sparsely recorded outside the nest, so that no model could be fitted. The predictors distance from the nearest nest and intranidal density were square root transformed. Density of RWA workers was incorporated either as a continuous (the square root of the number of workers) or a categorical factor (high density: > 20 workers, low density ≤ 20 workers) in separate models (i.e. two models per species). From the full model, we removed with the drop1 function fixed factors which removal did not significantly reduce the explanatory power of the model [56]. In addition, we fitted a similar generalized mixed model to explain total species richness (sum of all myrmecophile species) along the forest spatial gradient. Here we opted for a glmer rather than a glmer.nb as there was no overdispersion. RWA density, distance from the nearest nest and species richness of the nearest nest (square root transformed) were modelled as covariates, sampling period and nearest nest as random factors.

Second, we assessed whether the myrmecophile community preferentially moved along the shortest path to another nest (edge direction). Myrmecophiles travelling along the forest edge follow the shortest path to the nearest nest (the location of all nests is known), as all nests are lined along the forest edge (Fig. 1, Additional file 1, Fig. S1). Myrmecophiles caught in the edge pitfalls between two nests could originate from either of the adjoining nests when they were moving between these nests, whereas peripheral and forest pitfalls mainly capture myrmecophiles from the nearest nest (Fig. 1). To make the sampling effort of the forest pitfalls comparable with the edge pitfalls, we pooled the total number of species over the two inner forest pitfalls per pair of nests. As such, for each pair of nests, we obtained one data point with myrmecophiles caught in the forest and one along the edge at the same distance away from the nests (Fig. 1). Because of the positioning of the nests, the focus here is on nest pairs rather than individual nests. Sampling was replicated three times for each pair of nests. Note that we did not include the data of the peripheral pitfalls (at distance 0 m) in these analyses, as directionality of movement could otherwise not be tested.

We modelled the predictors directionality of movement (edge vs forest), distance to the nearest nest and density of RWA workers to predict the response variable species richness (total number of myrmecophile species) using a generalized linear mixed-effect model with Poisson error distribution and log link function. We also included the intranidal species richness pooled over a pair of nests as a fourth covariate. Pair of nests and sampling period were modelled as random factors. From the full model, we removed with the drop1 function fixed factors which removal did not significantly reduce the explanatory power of the model [56]. We performed LR-tests to assess the significance of the fixed effects in the reduced species richness model.

We validated all models by analyzing their residuals in the DHARMa package [57], but no issues were identified. Significance of the predictors was estimated with a χ^2^ Wald (type 3) test using the function Anova (car package).

### Colonization dynamics of myrmecophiles

To examine the colonization dynamics of RWA myrmecophiles, we compared the diversity and identity of supported myrmecophiles between well-established, mature nests (“old nests”) and newly founded nests (“new nests”). The distribution of RWA mounds in the study sites have been intensely surveyed for the last 20 years [49]. Therefore, we have a clear idea of the age of the mounds in these sites. We selected old (2 sites: OB: *N =* 4, WV: *N =* 8) nests which were older than 5 years (mean surface: 4.94 m^2^ ± SE 0.46). Newly founded nests (2 sites: OB: *N =* 8, WV: *N =* 7) arise during spring and were smaller (mean surface: 1.83 m^2^ ± SE 0.32). Sampling was during summer, so these nests were younger than half a year at the time of sampling (Fig. S2d). To avoid lasting damage to the small, new nests, we used non-invasive pitfalls in this experiment (Fig. S2e). They consisted of a plastic 0.5 L pot (height 7 cm) with a 1 cm plaster bottom and a top opening (diameter 11 cm). The pitfall was filled with wood chips (*Pinus maritima*, commercially available DCM bark). The myrmecophiles could enter the pitfall through the top opening or through four circular openings (diameter: 1.5 cm) that were made at 90° in the lower part of the pot. In contrast to the pitfalls used in the previous experiment, myrmecophiles were able to exit the pitfall and myrmecophiles were here thus not accumulated over time. We placed a pitfall deep inside the nest with the top rim level with the interface between the aboveground organic material mound and the underground earth nest. The pitfalls were left for 2 weeks in the nest and then checked for myrmecophiles in a large tray in the field. Afterwards, myrmecophiles were put back in the nest and the pitfall with wood chips was re-installed. Every nest was resampled four to six times, with a 14-days interval between each resampling. Sampling took place between the end of June and end of August, either in the summer of 2018 or 2019. Note that colonization here can occur through running on the ground, but also by flying or passive transport (see carrying of *Clytra quadripunctata* by the host [39]).

We constructed a negative binomial generalized linear mixed-effect model to predict total species richness in a nest as a function of the fixed effects nest age (old vs new), connectivity (the number of mature nests within a 100 m radius) and site (OB or WV). The first order interactions between the predictors were also modelled. Nest identity was included as a random variable as nests were resampled (4–6 times). From the full models, we removed terms using the drop1 function [56]. We validated this model by analyzing its residuals in the DHARMa package [57]. We did not identify residual problems.

All statistical analyses were performed using R (version 3.4.2).

## Results

### Spatial distribution of myrmecophiles outside the host nest

#### Myrmecophiles abundant outside host nest, but mobility is functional group-specific

**Fig. 2:**
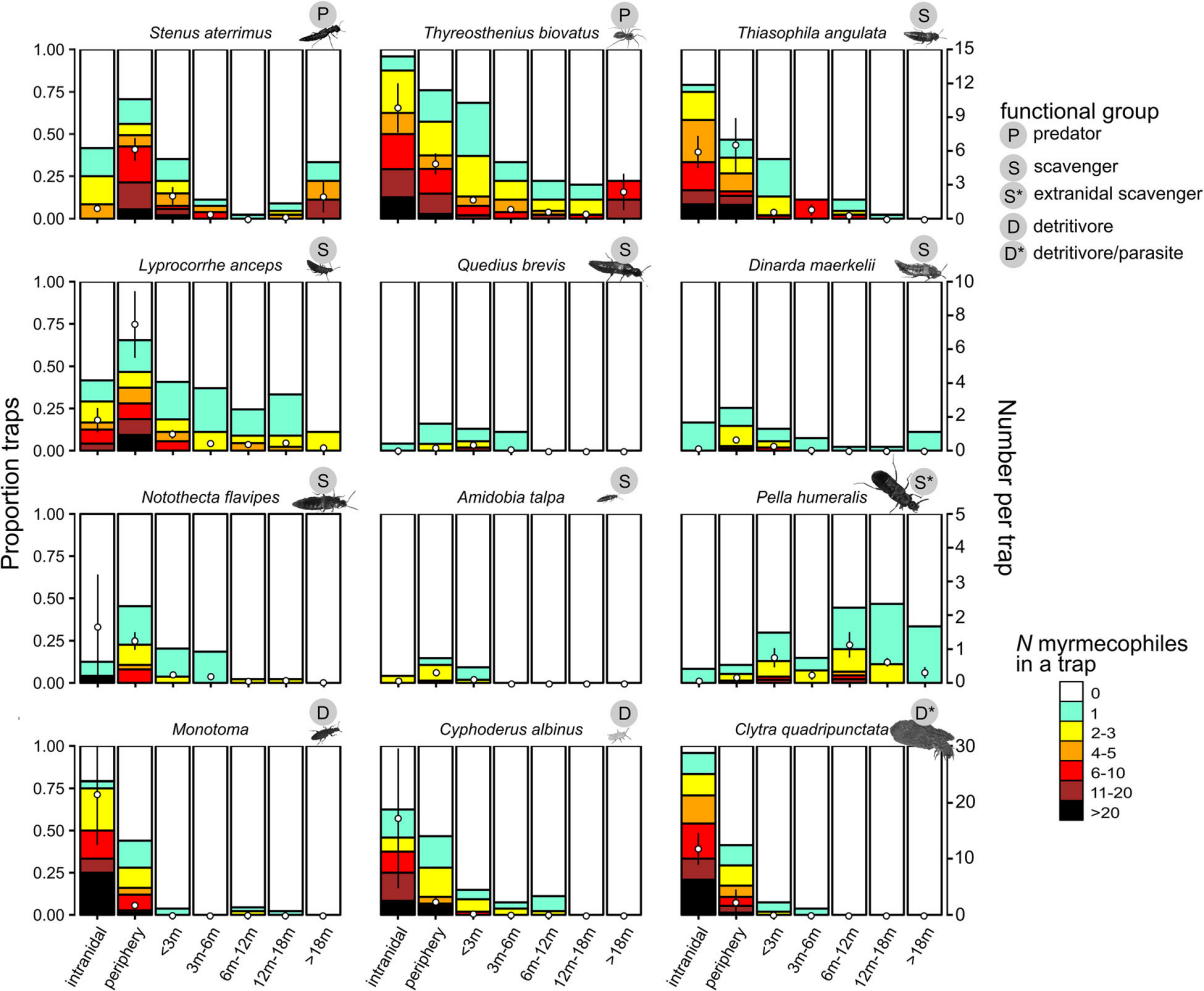
Spatial distribution of the 12 most widely distributed myrmecophile species in the community (present in more than 10 pitfalls). The pitfalls along the spatial gradient have been grouped in seven different distance bins: ‘intranidal’ (*N*pitfalls = 24), ‘periphery’ (0 m, *N*pitfalls = 75) and five distance bins of outside pitfalls (‘< 3 m’: Npitfalls = 54, ‘3 m–6 m’: *N*pitfalls = 27, ‘6 m–12 m’: *N*pitfalls = 45, ‘12 m–18 m’: *N*pitfalls = 45, ‘> 18 m’: *N*pitfalls = 9). For each distance bin, the proportion of pitfalls with 0, 1, 2–3, 4–5, 6–10, 11–20 and more than 20 individuals of a particular species is indicated with a multicolored stacked bar. The left y-axis shows the proportional distribution of these abundance classes along the distance gradient (x-axis). For each species, we also plotted the average abundance ± SE of individuals in a pitfall per distance bin with black-bordered circles. The y-axis corresponding to these average abundances is given on the right

We recorded 3436 obligate myrmecophiles belonging to 17 species (two *Monotoma* species were grouped) at the periphery and outside the nest of their *Formica rufa* host. The distribution of myrmecophiles was related to the functional role in the community. Predatory species and, to a lesser extent, scavengers were more mobile and had a higher tendency to reside outside the nest than detritivorous species. The spider *Thyreosthenius biovatus* and the beetles *Monotoma* and *Clytra quadripunctata* were present in most nests and reached high densities in the pitfalls (Fig. 2, Table 1). The rove beetles *Stenus aterrimus, Lyprocorrhe anceps* and *Notothecta flavipes* occurred in a higher percentage of pitfalls at the periphery than inside the nest (Fig. 2, Table 1). Most species were captured in a lower percentage of pitfalls and in lower abundances with increasing isolation from the host nest (Fig. 2), but this pattern was not present in the rove beetle *Pella humeralis*. This beetle was also atypical in the myrmecophile community as it almost exclusively occurred outside the nest.

**Table 1:** Distribution of the myrmecophiles in the pitfalls (WV site). For each myrmecophile species, the number of captured individuals (*N*_*ind*_) and number of pitfalls with at least one individual (*N*_*pitfall*_) are summarized for intranidal pitfalls (*N =* 24), pitfalls at the periphery (*N =* 75), and pitfalls outside the nests (> 0 m, *N =* 180)

Species	Functional group	Taxon	Intranidal (24 pitfalls)		Periphery (75 pitfalls)		Outside (180 pitfalls)		Total records
			*N*_*ind*_	*N*_*pitfall*_	*N*_*ind*_	*N*_*pitfall*_	*N*_*ind*_	*N*_*pitfall*_	
*Stenus aterrimus*	predator	Coleoptera (Staphylinidae)	22	10	467	53	152	30	641
*Thyreosthenius biovatus*	predator	Araneae (Linyphiidae)	238	23	370	57	189	67	797
*Thiasophila angulata*	scavenger	Coleoptera (Staphylinidae)	144	19	496	35	73	28	713
*Lyprocorrhe anceps*	scavenger	Coleoptera (Staphylinidae)	45	10	565	49	113	59	723
*Quedius brevis*	scavenger	Coleoptera (Staphylinidae)	1	1	16	12	24	10	41
*Dinarda maerkelii*	scavenger	Coleoptera (Staphylinidae)	4	4	52	19	22	12	78
*Notothecta flavipes*	scavenger	Coleoptera (Staphylinidae)	40	3	94	34	23	18	157
*Amidobia talpa*	scavenger	Coleoptera (Staphylinidae)	2	1	25	11	7	5	34
*Leptacinus formicetorum*	scavenger	Coleoptera (Staphylinidae)	1	1	0	0	1	1	2
*Myrmetes paykulli*	scavenger	Coleoptera (Histeridae)	2	2	3	3	1	1	6
*Pella humeralis*	extranidal scavenger	Coleoptera (Staphylinidae)	2	2	14	8	133	64	149
*Monotoma*	detritivore	Coleoptera (Monotomidae)	518	19	140	33	6	5	664
*Cyphoderus albinus*	detritivore	Collembola (Cyphoderidae)	416	15	184	35	27	15	627
*Spavius glaber*	detritivore	Coleoptera (Cryptophagidae)	0	0	5	2	0	0	5
*Platyarthrus hoffmannseggii*	detritivore	Isopoda (Platyarthridae)	9	1	51	3	0	0	60
*Clytra quadripunctata*	detritivore/parasite	Coleoptera (Chrysomelidae)	286	23	176	31	7	5	469

**Fig. 3:**
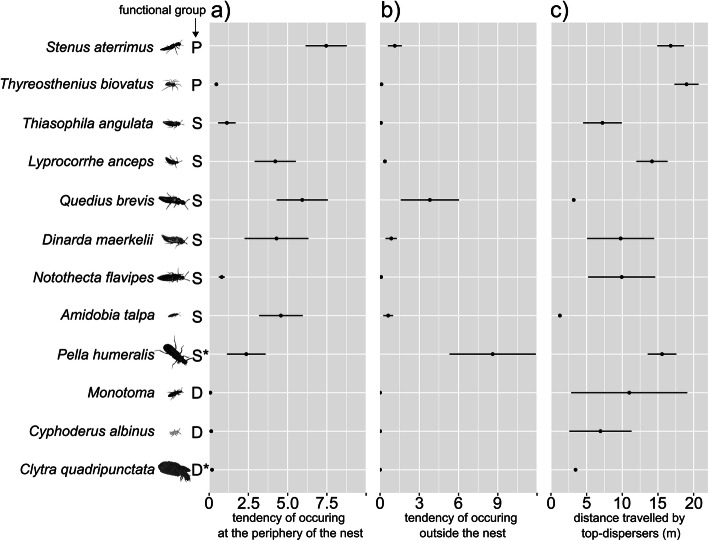
Tendency of myrmecophiles associated with red wood ants to leave the nest. (**a)** Tendency of occurring at the periphery of the nest (abundance in a peripheral pitfall/abundance in an intranidal pitfall) (**b**) Tendency of occurring outside the nest (abundance in an outside pitfall/abundance in an intranidal pitfall) (**c**) Mean distance travelled by the 10% top dispersers for each species. Functional groups: P predator, S scavenger, S* extranidal scavenger, D detritivore, D* detritivore/parasite. Error bars indicate standard errors. Post hoc differences see Additional file 2: Table S1–3

**Fig. 4:**
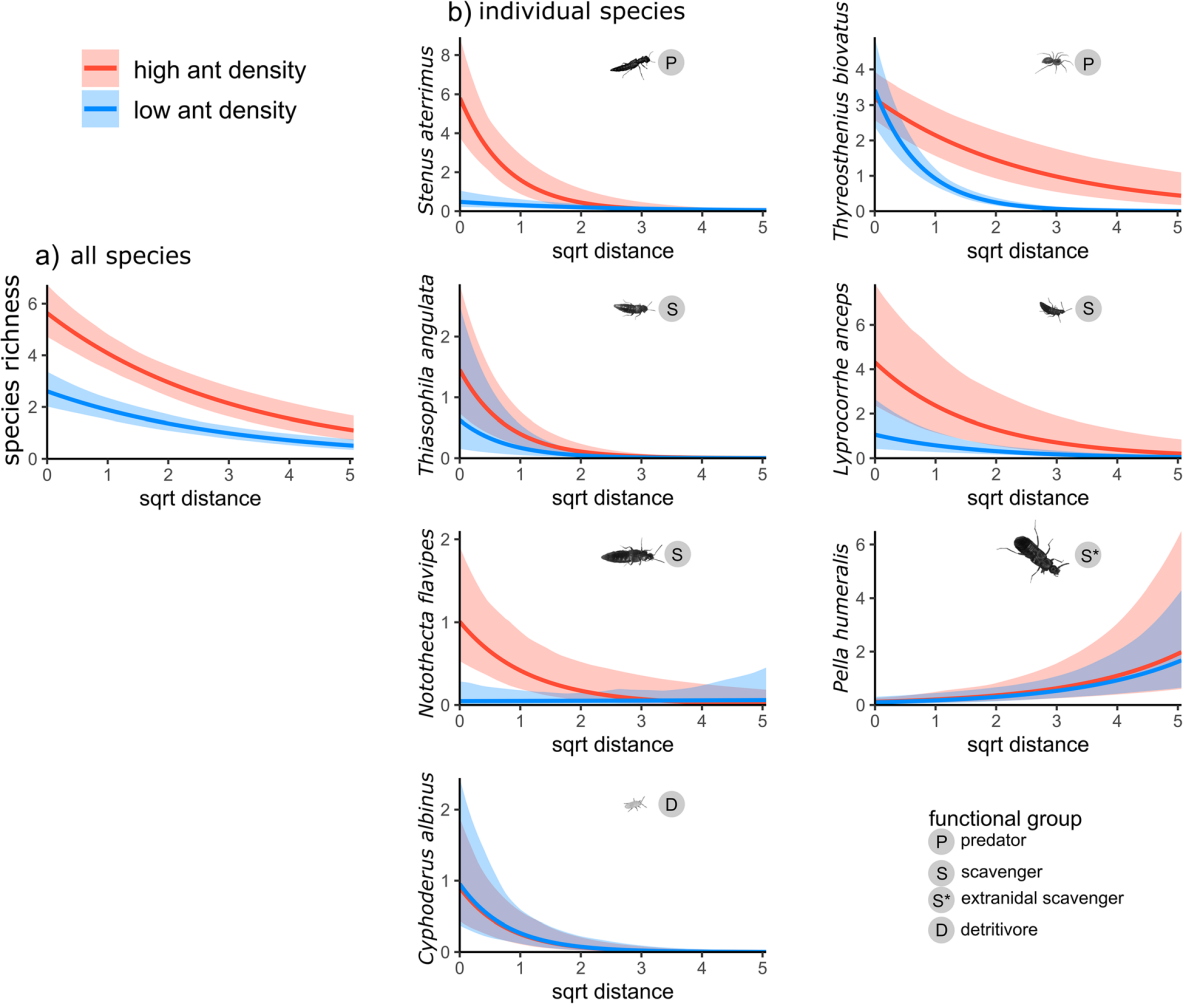
Effect plots corresponding to the mixed models in Table 2 (gradient towards forest interior and host density as categorical variable). The plots display the partial effect of distance away from the nest and host ant density (high density: > 20 workers, low density ≤ 20 workers) on myrmecophile distribution, while other predictors are held fixed: **a** total species richness with increasing distance from the nearest nest (**b**) the change in abundance for individual myrmecophile species with increasing distance from the nearest nest (± 95% CI, 100 bootstrap replicates). These plots are related to Fig. 2. However, Fig. 2 also includes data from edge pitfalls, does not account for other predictors and its x-axis gives the distance away from the nearest nest in distance bins rather than as a continuous variable

Myrmecophile species greatly differed in their tendency to occur at the periphery of the nest (Kruskal-Wallis test, chi-squared = 45.39, df = 11, *P* < 0.001, Fig. 3a, Additional file 1: Fig. S3, Post hoc differences Additional file 2: Table S1). *Stenus aterrimus* and *Q. brevis* tend to occur more often at the periphery than other species. Similarly, the average number of individuals in an extranidal pitfall divided by the average number of individuals in a nest pitfall was greatly different among the myrmecophile species (Kruskal-Wallis test, chi-squared = 54.705, df = 11, *P* < 0.001, Fig. 3b, Additional file 1: Fig. S3, Post hoc differences Additional file 2: Table S2). The detritivores *Monotoma*, *C. albinus* and *C. quadripunctata* had a very low tendency to leave the nest (Fig. 3b, Additional file 1: Fig. S3). *Pella humeralis* displayed the highest tendency to occur outside a nest (Fig. 3b, Additional file 1: Fig. S3). Myrmecophile species differed in the average distance travelled by the individuals at the upper 10% of their distance distribution (Kruskal-Wallis test, chi-squared = 79.83, df = 11, *P* < 0.001, Fig. 3c, Post hoc differences Additional file 2: Table S3). The predatory myrmecophiles *S. aterrimus*, *T. biovatus* had individuals that forage at a very large distance from host nests (Figs. 3c,4, Additional file 1: Fig. S3), whereas the detritivorous species *Monotoma*, *C. albinus* and *C. quadripuncta* only travelled low to moderate distances (Fig. 3c, Additional file 1: Fig. S3).

#### Co-movement of myrmecophiles and foraging red wood ants

Myrmecophile species richness decreased away from the host nest (Fig. 4a, Table 2). Myrmecophile species richness was higher when more ants were present at a given distance outside the nest (host density as a categorical or continuous factor in Table 2, as a categorical factor in Fig. 4a). This implies that the myrmecophile community prefers to co-move with foraging host workers. This co-movement was clearly present in the predatory species *T. biovatus* and *S. aterrimus*, and in the scavenging species *T. angulata*, *L. anceps* and *N. flavipes*, as their individual distribution was positively correlated with the distribution of the host workers outside the nest (Fig. 4b, Table 2). The density of the detritivorous springtail *C. albinus* outside the nest was not correlated with higher host densities. Unlike other myrmecophiles, the density of *P. humeralis* increased away from the nest (Fig. 4b, Table 2). The number of individuals/species found outside the nest positively correlated with the number of myrmecophilous individuals found in a nest (or number of species in case of the species richness model) in multiple models (Table 2). Finally, a higher number of species was found towards the inner forest than along the forest edge (Table 2, *P* < 0.001).

**Table 2:** Test results of the factors affecting spatial distribution outside host nests in the WV site (Type 3 Wald χ^2^ tests)

Response variable	model	predictor	Df	Host density continuous			Host density categorical (low vs high density)		
				effect	χ^**2**^	P	effect	χ^**2**^	P
Gradient towards the forest interior									
Total species richness	glmer	distance from nest	1	–	47.5	**< 0.001**	–	54.9	**< 0.001**
		host density	1	+	19.4	**< 0.001**	+	39.2	**< 0.001**
		number of species in the nest	1	+	6.5	**0.011**	+	10.2	**0.001**
		distance from nest x host density	1	+	9.1	**0.003**			
Number of *Thyreosthenius*	glmer.nb	distance from nest	1	–	36.5	**< 0.001**	–	10.2	**0.001**
		host density	1	+	3.9	**0.049**	+	0.0	0.84
		number of individuals in the nest	1	+	14.5	**< 0.001**	+	11.9	**< 0.001**
		distance from nest x host density	1	+	15.5	**< 0.001**	+	15.8	**< 0.001**
Number of *Stenus*	glmer.nb	distance from nest	1	–	25.7	**< 0.001**	–	33.5	**< 0.001**
		host density	1	+	16.0	**< 0.001**	+	26.2	**< 0.001**
		distance from nest x host density					–	11.8	**< 0.001**
Number of *Thiasophila*	glmer.nb	distance from nest	1	–	31.4	**< 0.001**	–	38.1	**< 0.001**
		host density	1	+	10.6	**0.001**	+	3.4	0.07
		number of individuals in the nest	1	+	11.4	**< 0.001**			
Number of *Lyprocorrhe*	glmer.nb	distance from nest	1	–	20.7	**< 0.001**	–	23.0	**< 0.001**
		host density	1	+	22.5	**< 0.001**	+	13.4	**< 0.001**
Number of *Notothecta*	glmer.nb	distance from nest	1	–	8.7	**0.003**	–	11.3	**< 0.001**
		host density	1	+	18.2	**< 0.001**	+	13.6	**< 0.001**
		number of individuals in the nest					+	3.2	0.072
		distance from nest x host density					–	5.6	**0.018**
Number of *Pella*	glmer.nb	distance from nest	1	+	24.9	**< 0.001**	+	24.9	**< 0.001**
Number of *Cyphoderus*	glmer.nb	distance from nest	1	–	36.0	**< 0.001**	–	36.0	**< 0.001**
		number of individuals in the nest	1	+	3.7	**0.06**	+	3.7	0.06
Gradient forest vs edge									
Total species richness	glmer	directionality of movement	1	–	14.7	**< 0.001**		6.1	**0.013**
		distance from nest	1	+	6.8	**0.010**	–	8.5	**0.003**
		host density	1	+	31.8	**< 0.001**	+	9.7	**0.002**

### Colonization dynamics of myrmecophiles

**Fig. 5:**
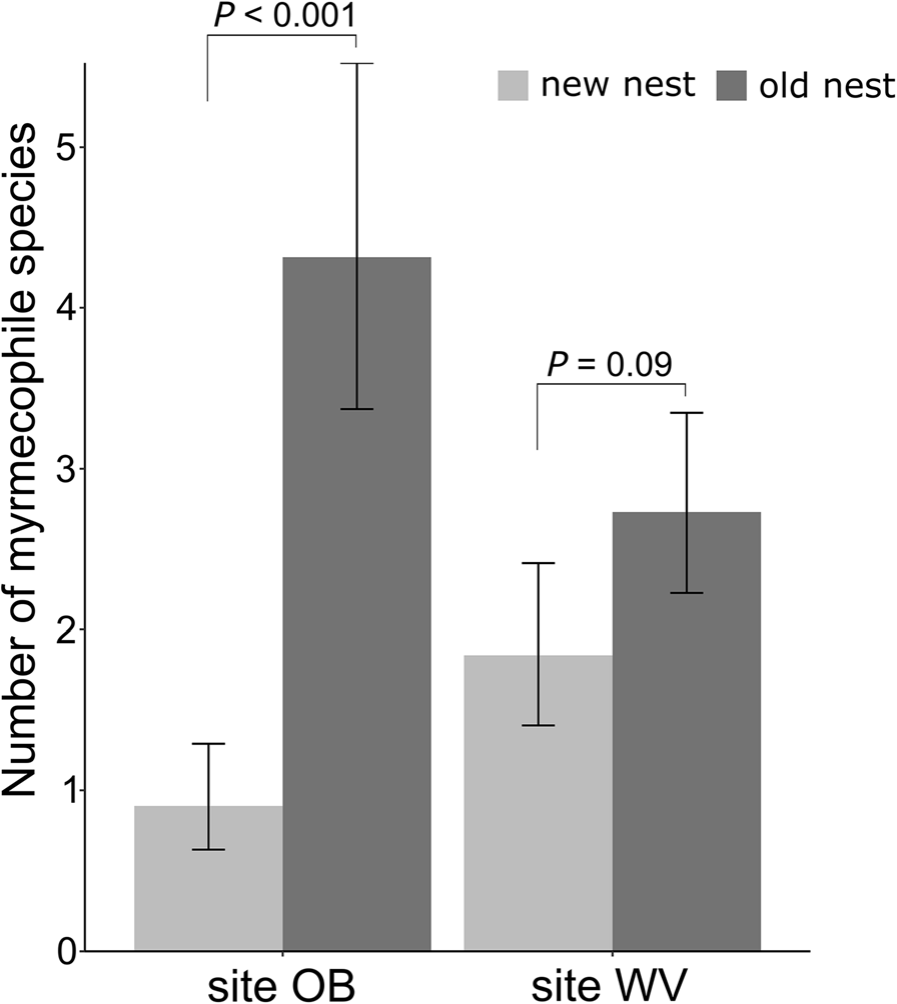
Effect plot showing the partial effect of nest age on species richness (± 95% CI) for site OB and site WV while the predictor connectivity in the model is held fixed. Old nests hold higher number of species than new nests for a given level of connectivity, but this effect was clearer in the OB-site

**Fig. 6:**
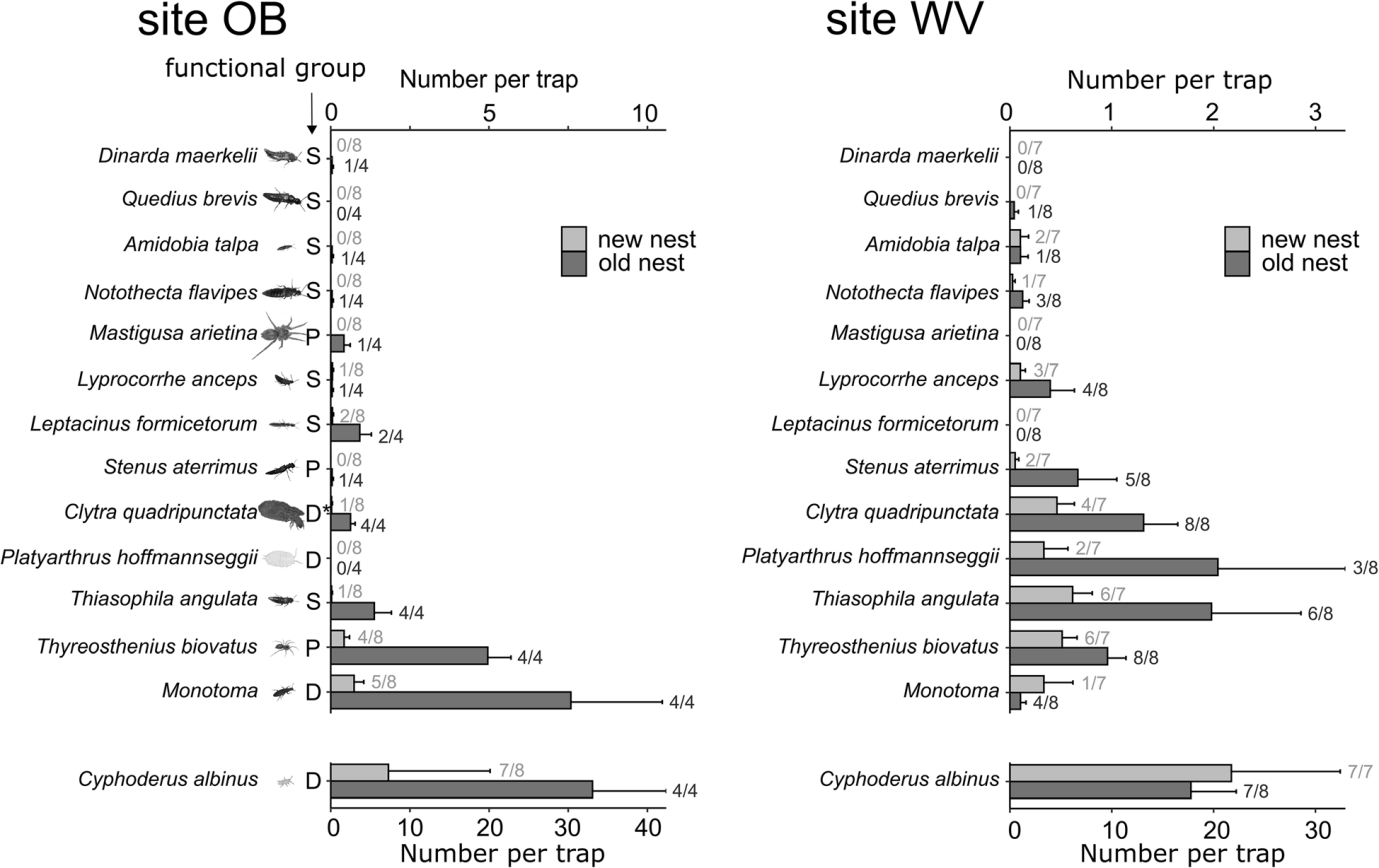
Average abundance ± SE of myrmecophile species found using wood chip pitfalls in new and old nests in the OB (*N*_new_ = 8 *N*_old_ = 4*)* and WV-site (*N*_new_ = 7 *N*_old_ = 8). *Cyphoderus albinus* average abundance per trap given on lower axis, abundances of other myrmecophiles given on the top-axis. Functional groups: P predator, S scavenger, D detritivore, D* detritivore/parasite. The proportion of new and old nests where the myrmecophile species was found at least once (each nest was sampled four to six times) is given to the right of the average abundance bars

Newly founded nests supported fewer myrmecophile species than old nests (glmer.nb, df = 1, χ^2^ = 50.3, *P* < 0.001, Fig. 5). The difference in number of species between old and new nests (OB site: Post-hoc Tukey test: *P* < 0.001; WV-site Post-hoc Tukey test: *P* = 0.09) was higher in the site OB than in the WV-site (Fig. 5). Nest connectivity positively affected species richness, both in new and old nests (glmer.nb, df = 1, χ^2^ = 7.8, *P* = 0.005). There was a lower likelihood to find myrmecophiles in new nests. The proportion of new and old nests colonized by each species is given in Fig. 6. The density of myrmecophile populations, and especially in the OB-site, was mostly lower in new nests (for each species, bar lengths proportional to mean abundance in Fig. 6). However, almost all myrmecophile species were able to colonize new nests in the first months after they were founded (Fig. 6). Only *Dinarda maerkelii*, *Quedius brevis* and *Mastigusa arietina* were not recorded in the new nests, but these species were also caught in very low numbers in old nests.

## Discussion

We found a remarkably high number of intranidal ant symbionts or myrmecophiles outside their host nest. We showed that these tightly connected ant symbiont communities are also connected during movement, by following the movement of their shared host. There was heterogeneous variation among symbiont groups which was linked to their functional role in the community. Assembly processes in new patches could not be directly connected to these differences in mobility.

The majority of ant species are central-place foragers which construct permanent nests [58]. Myrmecophiles obligately living in the nest of these ants are only sporadically reported outside the host nest [10, 36–40, 59] and are typically collected by opening the nest. Red wood ant (RWA) myrmecophiles of this study have been occasionally recorded outside the nest using pitfalls or hand capture: *T. angulata* [38, 60], *T. biovatus* [61–63], *P. hoffmannseggii* [64, 65], *N. flavipes* [66, 67], *S. aterrimus*: [67], *A. talpa* [67], *Q. brevis* [66], *M. paykulli* [68], but they have always been reported in very small numbers (max. five individuals) (cfr. their large densities inside RWA nests [69]). The large number of records outside the nest, and including all members of the studied community, here is therefore unexpected and very novel. The records of myrmecophiles associated with other permanent ant nests often seemed to be linked to specific events in the host colony life cycle (e.g. [10]). Some myrmecophile species were recorded when they followed their host colony moving to a new nest site [39]. Flying *Paussus* beetles are captured using light pitfalls and in increased numbers at the beginning of the rains, coinciding with the host’s nuptial flights [70]. The high extranidal mobility found in the RWA myrmecophile community, by contrast, was found all summer and probably spans from spring to autumn. It should be noted that high mobility is known in the peculiar group of myrmecophiles associated with nomadic army ants. These ants do not construct permanent nests and are almost incessantly on the move [71]. Consequently, there is a high selection pressure on the associated myrmecophile fauna to keep pace with the very mobile host colony. They mainly achieve this by running independently among the moving ants on the trails or by phoretic transport [71, 72].

Species in the myrmecophile community greatly differed in their tendency to exit the nest and the distance they travelled away from the host nest. The extranidal mobility was strongly correlated with their functional role. Detritivorous species were more restricted to the host nest than predatory species. Moreover, leaving individuals of detritivores stayed closer to the nest than those of predatory species. Differential mobility among competing species may result in a competition-colonization trade-off, which promotes the community assembly of regular metacommunities [73], but also of symbiont communities (e.g. [58]). However, species that compete for the same food sources in the myrmecophile system tend to have similar degrees of mobility. It is unclear whether the high mobility of predatory species is translated into superior dispersal capacities. It is possible that the predatory species leave the nest to hunt for prey and return. The rove beetle *Pella humeralis* showed a deviating spatial distribution. It was rarely found in or near the nest, but was the dominant myrmecophile at greater distances away from the nest. Other studies showed that this species and congeners frequently dwell around ant nests [58, 74].

Organisms move non-randomly in the landscape and they often prefer certain routes to move from one patch to another, as evidenced in insects [75], amphibians [76], birds [77] and mammals [78]. Likewise, the myrmecophile community associated with RWAs did show directed movement outside the nest. They preferentially moved along the highest density of ants outside the nest (such as along trails) and avoided the forest edge. Central-place foraging ants often deploy a network of pheromone trails radiating out to food sources [58], and this web of trails is especially well developed in RWAs [47, 79]. Lab experiments demonstrated that pheromone trails of ants may be followed by symbionts [41–45]. Here, we found that RWA myrmecophiles likely exploit these cues to co-move in the landscape in a natural setting. Running among large numbers of workers offers the myrmecophiles protection against predators. The RWA myrmecophiles can flexibly shift between foraging, dispersal or escaping from enemies as they do not co-move attached to a vector species. Ant trails may also guide myrmecophiles to extranidal food sources or lead them to new nests as trails may overlap or connect different nests [47]. The denser network of ant trails and the polydomous organization with inter-nest trails in the WV site may have resulted in a faster colonization of newly founded nests compared to the OB site. Movement was also directed away from the forest edges. These edges are characterized by higher temperature fluctuations, higher light levels, reduced moisture and increased predation [80]. The higher stress at the edge may explain the preferential movement of the myrmecophiles away from the edge.

The process of colonization and succession of new habitat patches (habitat islands) reveals how communities may adapt to fluctuating patch availability and assemble over time. Host-symbiont communities provide ideal microcosms to track colonization in natural settings [81]. We tracked for the first time colonization of newly emerged ant nests by symbionts. In line with theoretical and empirical studies, we found lower diversity in newly founded nests than in mature nests [82–84]. Most myrmecophiles were able to colonize a new nest within the first months, but the lower observed diversity indicate that the associated communities did not reach an equilibrium, yet. The weakly mobile myrmecophiles *C. albinus* and *Monotoma* beetles surprisingly colonized most new nests and even reached the highest densities of the newly assembled communities. This discrepancy between extranidal motility and colonization can be caused by different processes. A few myrmecophilous species, such as the springtail *C. albinus,* target other ant hosts scattered over the study site, as well. These species can use nests of other ant species as stepping stones to colonize new RWA nests. This process could explain why *C. albinus* was able to rapidly colonize even the most isolated new RWA nest (400 m away from the nearest RWA nest). Another explanation is that the densities of myrmecophiles in new nests do not reflect the number of successful colonization events. It is possible that a few colonizers may reproduce rapidly. Furthermore, high extranidal mobility as observed in *S. aterrimus* and *T. biovatus* may be linked to foraging rather than to dispersing events. Lastly, the community has other modes of dispersing than running. One species, the larvae of the beetle *Clytra quadripunctata*, may be carried by the host from one nest to another [39]. But more crucially, a large part of the community has functional wings. Flying has rarely been recorded in this community [49], and aerial dispersal is probably restricted to a narrow time frame in their life cycle or limited to particular seasonal conditions. This was also suggested by [38] who found that newly emerged *Thiasophila* beetles associated with RWAs were attracted to light and attempted to fly off. After 2 weeks, the beetles did no longer show attempts to fly, avoided light and mostly hid in the nest material. Overall, the relative importance of flying dispersal compared to dispersal by walking is unclear in this community.

## Conclusions

Future research may further elaborate this neat host-symbiont system and address fundamental ecological questions, such as assessing the relative role of local and regional processes in assembling metacommunities, and testing the effect of (co-)dispersal on the stability of the communities and food webs. Much theory on metacommunities and metafoodwebs were derived from the results of lab microcosms, but extending our focus to natural metacommunities, and in which the movement of a species might be directly or indirectly affected by other species, could start to fill the gap in our understanding of the dynamics of realistic metacommunities.

## Supplementary Information

**Additional file 1: Supporting Figs. S1, S2 and S3. **S1. Map of red wood ant nest distribution in site WV and site OB. S2: Overview of the sampling of the myrmecophiles. S3: Relative abundances of the 12 most widely distributed myrmecophile species along the spatial gradient.

**Additional file 2: Supporting tables Table S1-S3.** Listing the Post-hoc test results related to Fig. 3.

## Data Availability

Datasets can be accessed at https://github.com/tjparmen/Moving-apart-together-co-movement-of-a-symbiont-community-and-their-ant-host.
