# Supplementary material for: Moving apart together: co-movement of a symbiont community and their ant host, and its importance for community assembly
Source: Mov Ecol. 2021 May 21;9:25. doi: 10.1186/s40462-021-00259-5 (PMC8140472; doi:10.1186/s40462-021-00259-5)
Supplement: Supplementary file 2 — Additional file 1: Supporting Figs. S1, S2 and S3. S1. Map of red wood ant nest distribution in site WV and site OB. S2: Overview of the sampling of the myrmecophiles. S3: Relative abundances of the 12 most widely distributed myrmecophile species along the spatial gradient. [file 40462_2021_259_MOESM1_ESM.pdf]

Supporting figures

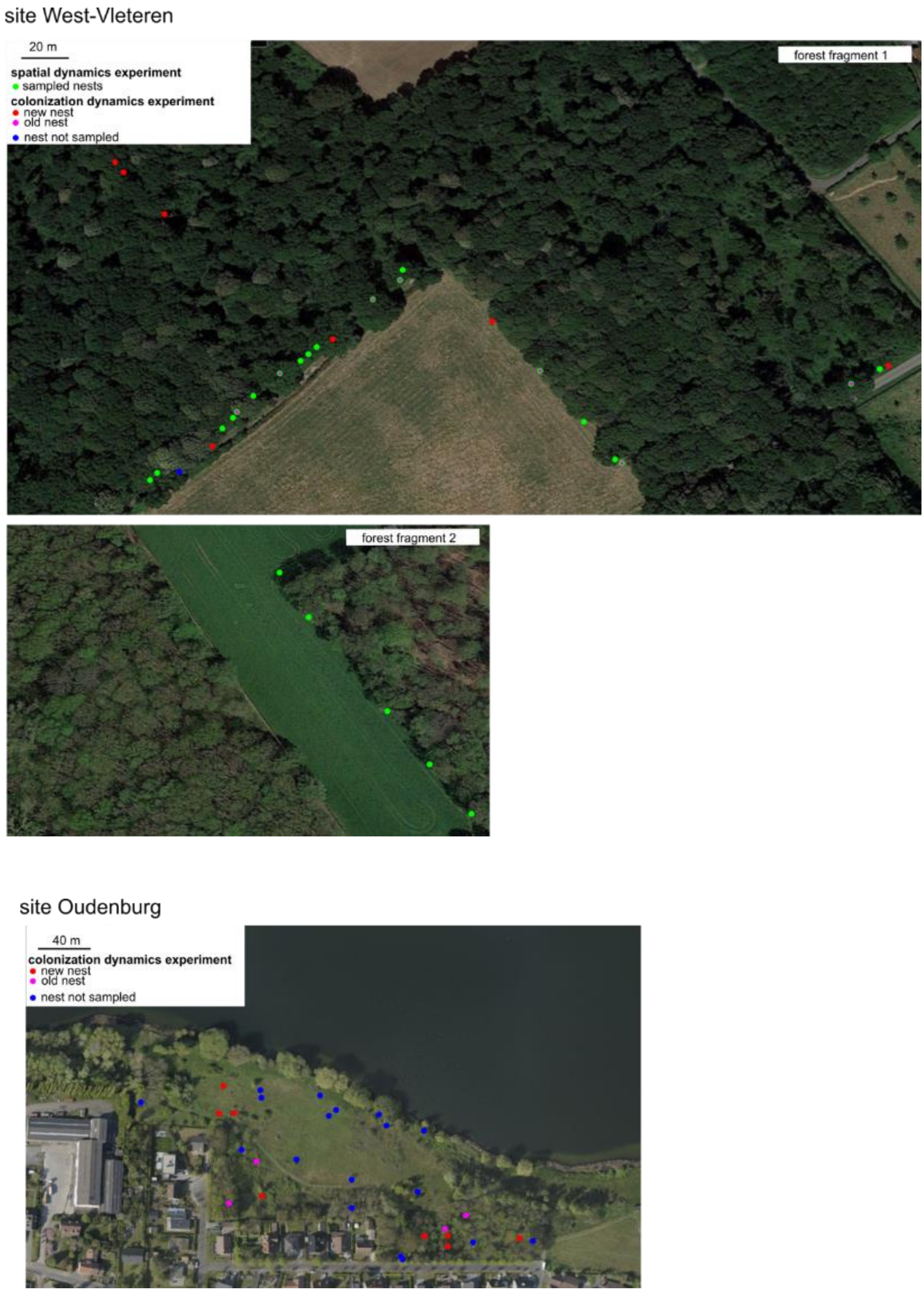

FIGURE S1. Map of red wood ant nest distribution. a) study site West-Vleteren (WV) with the sampled forest fragments: here both spatial distribution and colonization dynamics were tested b) study site Oudenburg (OB): here we only tested colonization dynamics.

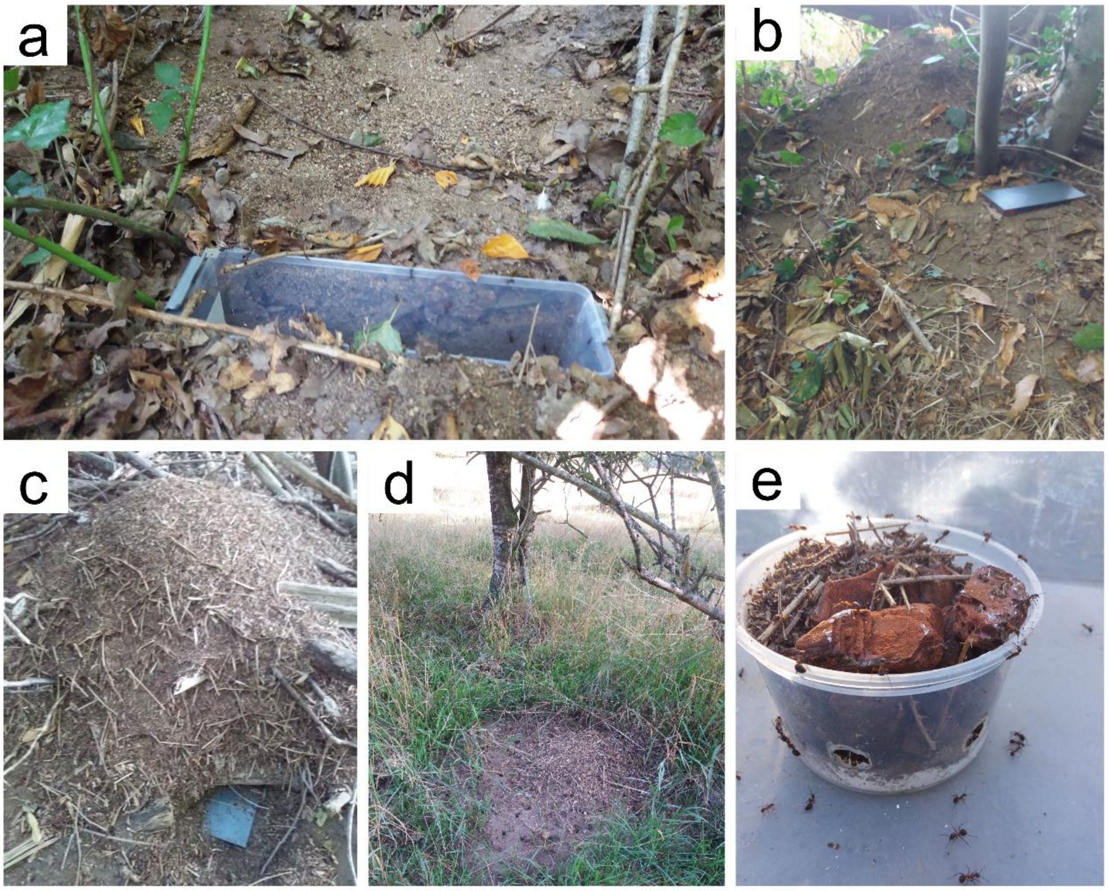

FIGURE S2. Overview of the sampling of myrmecophiles. a) rectangular “accumulation” pitfall to assess the spatial distribution of myrmecophiles outside the nest (here displayed without the plastic roof) b) Forest pitfall with grey roof outside red wood ant mound. c) Intranidal pitfall positioned in red wood ant mound to sample intranidal guests. The pitfall was completely covered with nest material afterwards. d) Newly founded red wood ant nest. e) Non-invasive pitfall with wood chips and a plaster floor used to sample myrmecophiles in the colonization dynamics experiment. Two of the four holes in the wall can be seen.

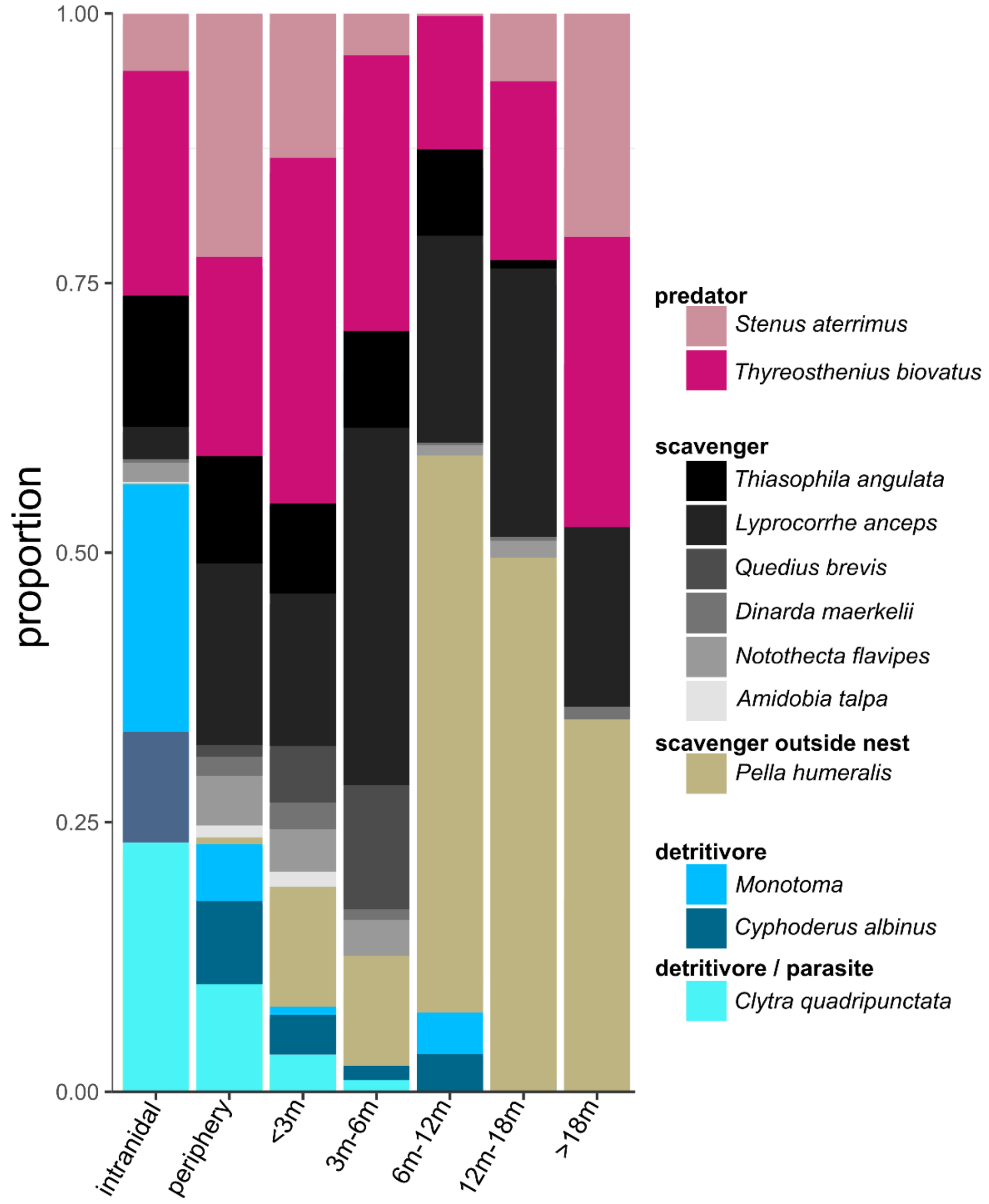

FIGURE S3. Relative abundances of the 12 most widely distributed myrmecophile species (present in more than 10 traps) along the spatial gradient.
