# Supplementary material for: Moving apart together: co-movement of a symbiont community and their ant host, and its importance for community assembly
Source: Mov Ecol. 2021 May 21;9:25. doi: 10.1186/s40462-021-00259-5 (PMC8140472; doi:10.1186/s40462-021-00259-5)
Supplement: Supplementary file 3 — Additional file 2: Supporting tables Table S1-S3. Listing the Post-hoc test results related to Fig. 3. [file 40462_2021_259_MOESM2_ESM.pdf]

Appendix S2. Supporting tables

Tendency of myrmecophiles associated with red wood ants to leave the nest: Post hoc tests. Letter codes refer to Tukey post-hoc test. Species with no letters in common are significantly different ($P < 0.05$).

Table S1. Tendency of occurring at the periphery of the nest with letter-based representation of Tukey pairwise tests.

Species	tendency	SE	letter code
Monotoma	0.1	0.0	e
Cyphoderus albinus	0.1	0.0	de
Clytra quadripunctata	0.2	0.1	ade
Thyreosthenius biovatus	0.5	0.1	a
Notothecta flavipes	0.8	0.2	acd
Thiasophila angulata	1.1	0.6	abcde
Pella humeralis	2.4	1.2	abcdef
Lyprocorrhe anceps	4.2	1.3	bcf
Dinarda maerkelii	4.3	2.0	abcdef
Amidobia talpa	4.6	1.4	bf
Quedius brevis	5.9	1.6	abcdef
Stenus aterrimus	7.5	1.3	f

Table S2. Tendency of occurring outside the nest with letter-based representation of Tukey pairwise tests.

Species	tendency	SE	letter code
Monotoma	0.0	0.0	c
Clytra quadripunctata	0.0	0.0	c
Cyphoderus albinus	0.0	0.0	c
Thiasophila angulata	0.1	0.0	ac
Notothecta flavipes	0.1	0.0	a
Thyreosthenius biovatus	0.1	0.0	ab
Lyprocorrhe anceps	0.4	0.1	d
Amidobia talpa	0.6	0.4	abcd
Dinarda maerkelii	0.9	0.4	abcd
Stenus aterrimus	1.1	0.5	bd
Quedius brevis	3.8	2.2	abcde
Pella humeralis	8.6	3.3	e

Table S3. Mean distance travelled by the 10% top dispersers for each species with letter-based representation of Tukey pairwise tests.

Species	distance (m)	SE	letter code
Amidobia talpa	1.3	4.1	de
Quedius brevis	3.2	2.4	d
Clytra quadripunctata	3.5	4.1	de
Cyphoderus albinus	6.9	2.2	bef
Thiasophila angulata	7.2	1.4	b
Dinarda maerkelii	9.8	2.4	bcdefg
Notothecta flavipes	9.9	2.4	bcef
Monotoma	11.0	4.1	acdefg
Lyprocorrhe anceps	14.2	1.1	c
Pella humeralis	15.6	1.0	ag
Stenus aterrimus	16.8	0.9	acfg
Thyreosthenius biovatus	19.0	0.9	a
